# Characterization of Retronasal Airflow Patterns during Intraoral Fluid Discrimination Using a Low-Cost, Open-Source Biosensing Platform

**DOI:** 10.3390/s22186817

**Published:** 2022-09-09

**Authors:** Graham A. Cousens, Michelle M. Fotis, Christine M. Bradshaw, Yida M. Ramirez-Alvarado, Christina R. McKittrick

**Affiliations:** 1 Department of Psychology, Drew University, 36 Madison Avenue, Madison, NJ 07940, USA; 2 Neuroscience Program, Drew University, 36 Madison Avenue, Madison, NJ 07940, USA; 3 Drew University, 36 Madison Avenue, Madison, NJ 07940, USA; 4 Department of Biology, Drew University, 36 Madison Avenue, Madison, NJ 07940, USA

**Keywords:** retronasal olfaction, deglutition, respiratory regulation, flavor perception, brain–computer interface

## Abstract

Nasal airflow plays a critical role in olfactory processes, and both retronasal and orthonasal olfaction involve sensorimotor processes that facilitate the delivery of volatiles to the olfactory epithelium during odor sampling. Although methods are readily available for monitoring nasal airflow characteristics in laboratory and clinical settings, our understanding of odor sampling behavior would be enhanced by the development of inexpensive wearable technologies. Thus, we developed a method of monitoring nasal air pressure using a lightweight, open-source brain–computer interface (BCI) system and used the system to characterize patterns of retronasal airflow in human participants performing an oral fluid discrimination task. Participants exhibited relatively sustained low-rate retronasal airflow during sampling punctuated by higher-rate pulses often associated with deglutition. Although characteristics of post-deglutitive pulses did not differ across fluid conditions, the cumulative duration, probability, and estimated volume of retronasal airflow were greater during discrimination of perceptually similar solutions. These findings demonstrate the utility of a consumer-grade BCI system in assessing human olfactory behavior. They suggest further that sensorimotor processes regulate retronasal airflow to optimize the delivery of volatiles to the olfactory epithelium and that discrimination of perceptually similar oral fluids may be accomplished by varying the duration of optimal airflow rate.

## 1. Introduction

In 1982, Paul Rozin proposed that “olfaction is the only dual sensory modality, in that it senses both objects in the external world and objects in the body (mouth)” [1]; indeed, subsequent studies have confirmed that olfaction involves the integration of two processes, with distinct perceptual sensitivities, anatomical substrates, and neural pathways [2,3,4,5]. Orthonasal processing plays a role in the detection of general environmental stimuli and involves the inhalation of odorants through the nares (nostrils) and activation of receptors in the olfactory epithelium in the nasal cavity. In contrast, retronasal olfaction is preferentially activated by odorants related to food [2,6] and involves the passage of volatiles from food in the mouth to the olfactory epithelium via the pharynx. In addition, the sensory pathways involved in retronasal processing have been shown to converge with those of the gustatory system within the brainstem to contribute to the perception of “flavor” in food (reviewed in [2,7]).

Both orthonasal and retronasal olfaction involve motor processes that facilitate the delivery of odorants to the sensory epithelium in the nasal cavity. Although volatile odorants can reach the olfactory epithelium orthonasally via respiratory inhalation, sniffing optimizes the activation of olfactory receptor neurons for odor detection and discrimination [8,9]. Variation in the volume, rate, duration, and pattern of airflow during sniffing is influenced by odorant composition [10], and the rate of sniffing may influence the deposition of odorant molecules within the olfactory epithelium in an odorant-specific manner [11]. Patterns of oscillatory local field potential (LFP) activity within the main olfactory bulb (MOB) and downstream cortical targets are coupled to the respiratory cycle [12,13], and the activity of individual mitral/tufted cells within the MOB is modulated by the frequency of the sniff cycle [14,15]. Further, LFP activity recorded within the MOB is coupled to diaphragmatic electromyographic (EMG) activity within respiratory and sniffing frequencies [16]. Thus, evidence suggests that sensory processes occurring within the olfactory system are associated with cyclical variations in airflow imposed by motor systems regulating respiratory and sniffing activity.

In contrast to orthonasal olfaction, relatively little is known about motor processes influencing nasal airflow during retronasal olfaction. Volatiles present in consumed foodstuffs are transported to the nasal cavity retronasally through the motor processes of mastication and deglutition [17,18,19,20], but to date, patterns of nasal airflow during food consumption have not been thoroughly investigated. Indeed, a body of evidence suggests that the taste, odor and texture of foods all affect the initiation and temporal pattern of swallowing (reviewed by [21]), which, in turn, would affect the retronasal delivery of odorants during post-deglutitive exhalation.

Although methods such as spirometry are readily available for directly monitoring nasal airflow characteristics in laboratory and clinical settings, our understanding of odor sampling behavior, particularly in relation to food consumption, would be enhanced by the development of inexpensive, wearable technologies that can be utilized in everyday settings. Thus, we developed a novel approach for monitoring odor sampling behavior using a commercially-available biosensing system (Cyton biosensing board, OpenBCI, Brooklyn, NY, USA) coupled with a MEMS-based pressure transducer for measuring intranasal air pressure. The OpenBCI platform supports the recording of low-frequency physiological signals, including electroencephalographic (EEG) and EMG signals [22], as a foundation for brain–computer interface (BCI) applications. We evaluated the utility of this approach in a study aimed at determining whether variation in the volatile composition of intraoral fluid samples influences patterns of retronasal airflow. Our primary predictions were that participants’ respiration patterns would adjust to optimize the timing of retronasal airflow during the period immediately following oral contact with fluid samples and that retronasal airflow rates would be more pronounced during discrimination of fluids with similar aroma profiles, given the likely recruitment of retronasal olfactory processes for accurate performance.

## 2. Materials and Methods

### 2.1. Participants

A convenience sample of 20 participants, 18 years of age or older, was recruited by word of mouth. Data are reported here for 17 participants (10 female; median age 34 years, range 19–58 years) for whom a single, 60-trial session was completed. Exclusion criteria made known to the participants at the time of informed consent were self-reported history of smoking; self-reported smell or taste disorder; underlying health conditions associated with a higher risk of complications associated with COVID-19; history of heart disorders, such as congenital heart defects, coronary heart disease, or arrhythmia. Procedures were conducted in accordance with The Declaration of Helsinki and were approved by the Drew University Institutional Review Board.

### 2.2. Apparatus

**Figure 1:**
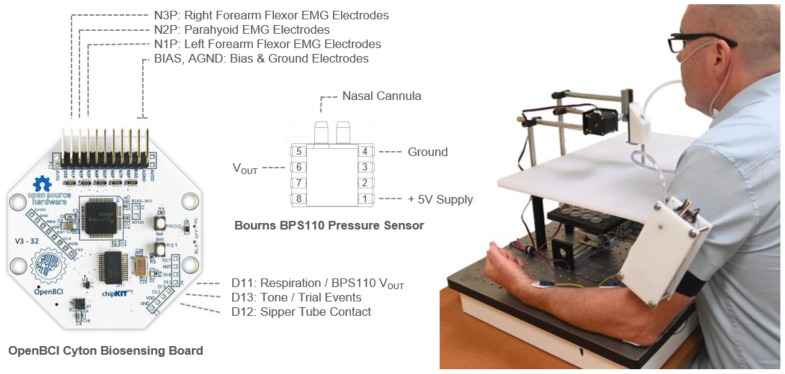
System components and experimental setup. (Left) OpenBCI Cyton biosensing board (image used with permission) and Bourns BPS110 differential pressure transducer. (Right) Behavioral testing equipment and wearable biosensing system. Forearm flexor behavioral responses and swallowing were monitored by surface electrodes connected to low-noise analog-to-digital converter input pins (ADS1299 N1P, N2P, N3P). Intranasal air pressure was monitored through a nasal cannula connected to one port of the pressure transducer, with output voltage sent to one of three general-purpose analog inputs (D11). Two additional analog inputs (D12, D13) were used to monitor trial events (tones) and mouth contact with fluid via the sipper tube. The BCI system was housed within an acrylic frame, which also contained a separate microcontroller (Arduino Uno) and battery pack for system expansion and power. Physiological signals were sent from the Cyton board via Bluetooth to a laptop computer (not shown) in an adjacent room.

Fluid samples consisted of two undiluted fruit juices (Fruit Punch and Triple Berry, Organic Juic’d Right, Wegmans Food Markets, Rochester, NY, USA; referred to as S1 and S2, respectively) or distilled water. These juices were selected because they contained similar sugar (62.5 g/L) and sodium (0.15 g/L) concentrations, were perceptually similar, and were readily available. As shown in Figure 1, samples were presented using a custom-built, automated linear motion platform loaded with a tray of thirty 20 mL glass scintillation vials. Oral fluid sampling was accomplished by sipping fluid through a polyethylene tube inserted into a stainless-steel sipper-tube assembly, which was lowered into each vial using a vertically positioned linear actuator. Movement of the platform and the assembly was coordinated by a microprocessor (Arduino Uno Rev3, Arduino, Somerville, MA, USA) operated by an experimenter in an adjacent room. To minimize passive exposure to odorants, the entire apparatus was housed within a partially enclosed chamber.

### 2.3. Procedures

Air pressure was measured continuously within the nasal vestibule using a disposable nasal cannula (flared tip Model 1108, Central Infusion Alliance, Skokie, IL, USA) connected via a 0.5 M length of flexible polyvinyl chloride tubing to a MEMS-based differential pressure transducer (BPS110, Bourns, Inc., Riverside, CA, USA). See Figure 1 for an overview of equipment and procedures. Consistent with other reports [20,23,24], pilot experiments showed that this method was a more sensitive and reliable measure of respiration patterns than other methods, such as nasal air temperature or thoracic stretch.

Characteristics of the pressure transducer, including a sampling rate (200 Hz) and pressure range (0.15 to 1.0 psi) suitable for human respiratory signals as well as electrical characteristics (5 V DC, 2 mA typical supply current; 1.9 mA typical output current) and physical properties (1.307 g; 12.7 mm width) suitable for integration with the microprocessor and the Cyton biosensing board, were perceived as benefits over other methods of assessing respiratory patterns. As noted by Hirst et al. [23], air pressure is a direct indicator of airflow, and the pressure transducer’s linear pressure–voltage transfer function allows estimation of relative differences in nasal airflow rate and volume across a range of voltages. However, given that the nasal cannula did not form a seal within the nasal cavity, estimations of absolute airflow rate or breath volume were not possible.

EMG signals associated with deglutition were recorded differentially using a pair of passive adhesive surface electrodes (Model 2560, 3M Healthcare, St. Paul, MN, USA) self-positioned by the participant approximately 4 cm apart on either side of the hyoid bone. This placement enabled clear identification of swallowing events but did not permit differentiation of activity of particular muscle groups (e.g., suprahyoid or infrahyoid) or phases of swallowing (see [25]), so EMG activity is referred to here as parahyoid. Additional pairs of electrodes were positioned approximately 10 cm apart on both the left and right forearms to monitor forearm flexor behavioral responses, and ground and bias electrodes were positioned on the left and right elbows. All signals were digitized and amplified (250 Hz; 24×) and sent via Bluetooth to a processor running OpenBCI acquisition software for data visualization, acquisition, and storage. Voltage values for EMG and respiration data were not referenced to an external standard and are thus expressed below as arbitrary voltage units.

Participants performed a three-choice oral fluid discrimination task while nasal air pressure and EMG activity were monitored. Prior to behavioral testing, participants were allowed to freely sample solutions and water from 250 mL stemless wine glasses to develop familiarity with the three fluid samples. Each session consisted of two blocks of 30 trials, and each trial initiated immediately after the automated lowering of the sipper-tube into a fluid vial. After a 7.5 s baseline recording period, a pure tone (0.5 s, 4 kHz) was presented, cueing the participant to sip fluid. The timing of sips was assessed via an analog channel of the biosensing board connected to the sipper-tube and grounded upon fluid contacting the mouth. The order of fluid samples followed a pseudorandom sequence which varied across blocks but was consistent across participants. Participants were instructed to remain as still as possible except when responding in order to minimize the occurrence of electrical artifacts and to breathe normally through the nose for the duration of the session. To encourage stimulus comparison and permit evaluation of perceptual differences across samples, participants were instructed to make a fist with either their left or right hands to indicate which of the two juice solutions, if either, were present in a given trial or to make a fist with both hands to indicate the presence of water. Forearm flexion was monitored continuously, and EMG voltage values were stored along with other physiological data. Handedness was not assessed. Participants were given verbal feedback on performance after each of the first eight trials of the first block to encourage consistent intake behavior and accurate identification of solutions during the remaining trials, and all data presented below are from the 52 uncoached trials occurring in each session.

### 2.4. Data Analysis

Initial signal processing was performed using Spike2 software (Cambridge Electronic Design, Cambridge, UK). EMG signals were digitally bandpass filtered (65–125 Hz) and converted by a root mean square function (RMS; 40 ms time constant). Event markers were created at the point of tone onset, fluid contact, and initial deflection of forearm EMG responses and stored along with filtered records of parahyoid EMG and raw, unfiltered records of nasal air pressure. Subsequent processing was conducted using custom scripts written in Matlab (The MathWorks, Natick, MA, USA).

Parahyoid EMG signals were smoothed with a variable Gaussian window (10–100 ms), and minimum amplitude (1–5 voltage units) and prominence (2–10 voltage units) of local maxima were adjusted for each participant’s data to optimize peak detection. Parahyoid EMG activity was typically minimal during baseline recording prior to tone onset but increased shortly thereafter as the participant moved toward and made contact with the sipper tube and drew fluid into the mouth. Our supposition was that the session-wide mode of the respiration signal voltage reflected the lack of inward or outward airflow associated with deglutition apnea during the pharyngeal phase of swallowing. This assumption was validated by visual inspection of EMG voltage traces during deglutition, which enabled the identification of local peaks of the parahyoid EMG signal occurring concurrently with stagnation of the respiration signal at the mode. Thus, we defined deglutition apnea as an interval of continuous respiration voltage within a tolerance of 1.5 voltage units of the session mode and containing at least one sample concurrent with a local peak of the parahyoid EMG signal.

Nasal air pressure voltage values below the mode were taken to reflect inward airflow and values above it to reflect outward airflow. To estimate the magnitude of post-deglutitive retronasal airflow across oral fluid conditions, we extracted respiration traces occurring immediately after the first occurrence of deglutition apnea in each trial, each trace consisting of a continuous sequence of voltage values beginning with the first value exceeding the mode (outward airflow) after deglutition apnea and ending with the first falling crossing of the mode (inward airflow). In addition, we extracted traces during a behavioral response interval between fluid contact and initial deflection of the forearm EMG signal since this interval corresponds to the time course of the perceptual decision in each trial. We calculated the total duration of retronasal airflow during these intervals, and given the ideal linear relationship between air pressure and the voltage output of the sensor, we estimated peak airflow rate as the maximum voltage and airflow volume as the area between the extracted waveform and the mode.

We calculated the probability of retronasal airflow in water, S1, and S2 trials during a 12 s interval centered on initial fluid contact. Probability functions were created for each stimulus type by calculating the proportion of trials at each 4 ms time point (3000 samples at 250 Hz) with voltage values greater than the session mode, indicating retronasal airflow. Given that the total number of correct water, S1, and S2 trials was similar (*n* = 168, 157, and 152 trials, respectively), and to track probability functions over time and between oral fluid conditions, we compared the three functions to a 2 s baseline period beginning 6 s prior to fluid contact in water trials. In order to identify significant deviations from this baseline, we created a sampling distribution of 100,000 probabilities, each calculated as the mean proportion of 150 randomly sampled voltage values greater than the session mode from a sampling frame of 84,000 samples (250 samples/s × 2 s × 168 correct water trials) occurring during the baseline period. The constructed probability distribution was normally distributed (Shapiro–Wilk, *p* > 0.05), and probabilities deviating from the distribution mean by >2.576 standard deviations (99% confidence interval) for >20 ms (five consecutive samples) were considered statistically significant.

Statistical analyses were conducted using SPSS (IBM Corp., Armonk, NY, USA). The normality of data was assessed using Shapiro–Wilk tests with alpha set to 0.05, and outliers were assessed using Grubbs tests [26] with alpha set to 0.025. Given the occurrence of non-normal distributions of several behavioral and physiological measures, we conducted nonparametric analyses on all data. Friedman tests were conducted to assess the main effects of oral fluid conditions, and Wilcoxon signed-rank tests were conducted post hoc, when appropriate, with alpha set to 0.017 using the Bonferroni correction method for multiple comparisons between fluid conditions. Confirmatory analyses conducted using parametric tests are not reported but yielded similar conclusions.

## 3. Results

### 3.1. Behavioral Performance

Thirteen of sixteen participants completed the session with a median accuracy of 94.23% correct trials and median response latency of 2.76 s in correct trials. Despite accurate discrimination performance overall, participants took longer to complete trials involving juice solutions and were less accurate in responding during those trials. While oral cavity cues were likely sufficient for rapidly discriminating water from the perceptually similar juice solutions, retronasal transport of volatiles contributed to accurate discrimination of the perceptually similar juice solutions (see Supplementary Materials for behavioral data).

### 3.2. Respiration and Deglutition

The system provided a reliable method for remotely monitoring intranasal air pressure and parahyoid EMG signals during the performance of the oral fluid discrimination task. Recordings from 2 of 13 participants for whom physiological data were collected contained periods with dropped respiration signals during the recording session and were, therefore, excluded from the analysis. Respiration data from an additional participant was not analyzed due to a pattern of clear oral breathing during the session. Thus, physiological data were analyzed for 10 participants.

**Figure 2:**
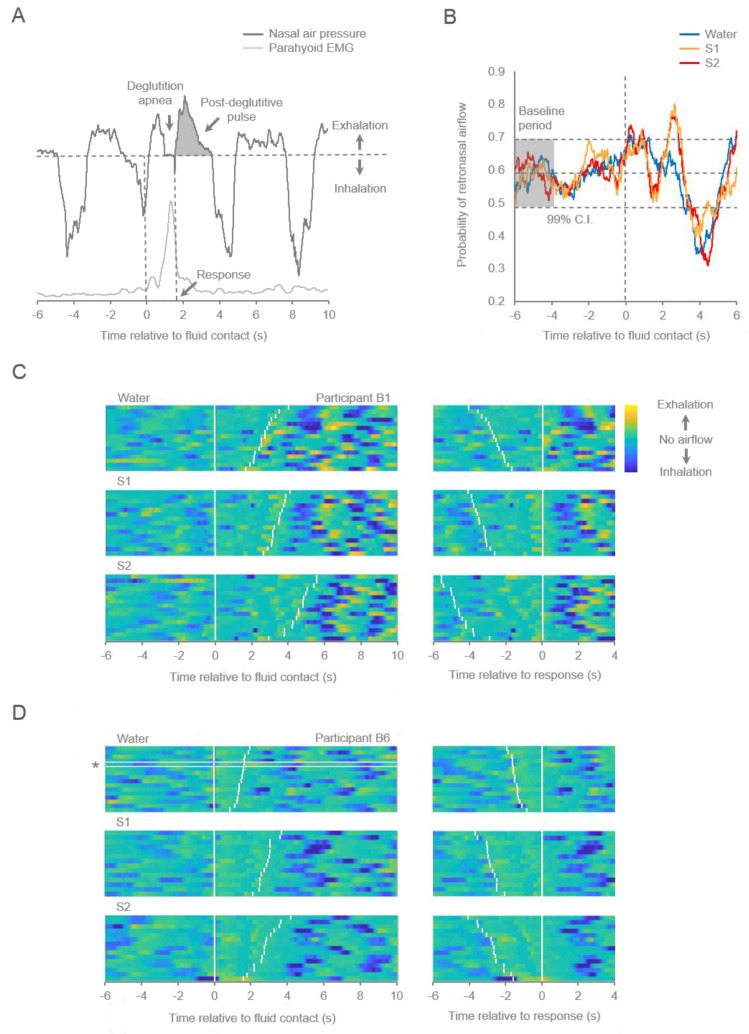
Nasal airflow during performance of the oral fluid discrimination task. (**A**) Illustrative nasal air pressure and parahyoid EMG traces recorded during a single trial with water as the sampled fluid. The horizontal dashed line represents the absence of airflow (session-wide mode). The vertical axis reflects voltage in arbitrary units of magnitude. Shaded region shows post-deglutitive airflow. (**B**) Probability of retronasal airflow for water, S1, and S2 trials. Middle horizontal dashed line represents the mean of a baseline period (−6 to −4 s) sampling distribution derived from 84,000 samples occurring across all correct water trials; upper and lower dashed lines represent 99% confidence intervals of this sampling distribution. See Section 2 for details. (**C**,**D**). Representative contour plots of nasal air pressure for two participants (B1, B6) across all correct water, S1, and S2 trials. Vertical axis reflects trials, which are sorted according to response latency. The horizontal axis reflects time (s) relative to fluid contact (left panels) or response initiation (right panels) in each trial. White event markers reflect the time of occurrence of fluid contact (left marker) and the behavioral response (right marker) in each trial. The magnitude of retronasal (exhalation) or orthonasal (inhalation) airflow is reflected by color intensity normalized for each participant, with the minimum voltage value pinned to the lowest color bar value (dark blue) and the session-wide mode voltage (no airflow) pinned to the middle color bar value (teal). * Identifies the trace illustrated in Panel (**A**).

As illustrated in Figure 2, we were able to detect typical patterns of respiratory airflow reflected in regular cycles of inward and outward airflow interrupted by bouts of apnea associated with deglutition (Figure 2A). Apnea occurred at least once between 0.5 and 6.0 s after fluid contact in 469/477 (98.32%) correct trials across all participants. As shown in Table 1, Friedman tests revealed a significant main effect of fluid type on the duration (*χ2* = 12.2, *p* = 0.002) but not latency (*χ2* = 4.2, *p* = 0.112) of deglutition apnea. Post hoc tests revealed significantly shorter apnea bouts in both S1 trials (*Z* = −2.803, *p* = 0.005) and S2 trials (*Z* = −2.599, *p* = 0.009) relative to water trials but no difference in apnea duration between S1 and S2 trials (*Z* = −0.153, *p* = 0.878). Inspection of these data revealed that one participant exhibited noticeably longer apnea bouts (median 1.51 s) compared to other participants (median 0.61 s), outlying the sample (*T*_10_ = 14.68, *p* < 0.001). However, since the inclusion of data from this participant had no impact on the pattern of findings or conclusions, these data were not omitted.

### 3.3. Retronasal Airflow

In addition, the system was sensitive enough to permit the evaluation of fine patterns of outward airflow during stimulus sampling and evaluation. We predicted that participants would time respiration to optimize the delivery of volatiles to the nasal cavity, with an increased likelihood of retronasal airflow during the interval between oral fluid contact and the behavioral response. As illustrated in Figure 2B, the probability of retronasal airflow during the 6 s interval prior to fluid contact did not differ from the baseline mean probability (0.591; see Section 2). However, this probability was elevated for all three fluid conditions for at least one interval within 1.30 s after fluid contact. Probability functions for S1 and S2 trials, but not water trials, were again significantly elevated between approximately 2.25 and 3.00 s after fluid contact. A comparison of probability functions to behavioral response times revealed that the median response latency in water trials (1.84 s) preceded the later interval and that latencies in S1 (3.01 s) and S2 trials (3.24 s) precisely followed it.

Consistent with previous reports (e.g., [23]), a prominent retronasal air pulse, or “swallow breath,” was typically observed immediately following deglutition (Figure 2A), occurring after the first apnea bout of each trial in 447/469 (95.3%) cases. However, as shown in Table 1, Friedman tests failed to reveal the significant main effects of the oral fluid condition on the duration (*χ2* = 0.8, *p* = 0.670), peak rate (*χ2* = 4.2, *p* = 0.112), or volume (*χ2* = 0.8, *p* = 0.670) of airflow during post-deglutitive pulses. Although post-deglutitive airflow is likely significant in conveying volatiles to the olfactory epithelium during ingestion, behavioral responses preceded the pulse onset in 183/469 (39.02%) trials. While this proportion of such cases was greatest for water trials (115/166, 69.28%), a substantial proportion occurred in S1 (39/154, 25.32%) and S2 (29/149, 19.46%) trials, demonstrating that pre-deglutitive airflow contributed to and was often sufficient for accurate perceptual discrimination.

Indeed, an inspection of respiration patterns across trials revealed that post-deglutitive air pulses were one component of a pattern of retronasal airflow initiated near the time of fluid contact and continuing for several seconds thereafter (Figure 2C,D; Supplementary Figure S2A–H). This period was characterized by an envelope of relatively sustained low-amplitude retronasal airflow typically beginning shortly after fluid contact in each trial and during which one or more retronasal pulses occurred. Post-deglutitive pulses occurred reliably following bouts of deglutition apnea, but pre-deglutitive airflow was also present in most trials. Further, multiple apnea bouts were common, especially in trials with longer response latencies. For 5/10 participants (Figure 2C; Supplementary Figure S2B–D,H), retronasal pulses occurring during fluid sampling had noticeably lower peak airflow rates compared to outward airflow occurring during normal respiration that resumed after behavioral responding, and for 1/10 participants (Supplementary Figure S2G), retronasal airflow during fluid sampling was noticeably higher than during respiration.

For most participants, the pattern of retronasal airflow observed during fluid sampling in S1 and S2 trials differed substantially from that observed in water trials. As shown in Table 1, Friedman tests revealed significant main effects of oral fluid conditions on the cumulative duration (*χ2* = 16.8, *p* < 0.001) and volume (*χ2* = 12.6, *p* = 0.002) of retronasal airflow, but not on peak airflow rate (*χ2* = 1.4, *p* = 0.497), during the interval between fluid contact and behavioral responding. Post hoc tests demonstrated that the cumulative duration of retronasal airflow was significantly greater in both S1 trials (*Z* = −2.803, *p* = 0.005) and S2 trials (*Z* = −2.803, *p* = 0.005) relative to water trials, while no difference was observed between S1 and S2 trials (*Z* = −2.191, *p* = 0.028). Similarly, airflow volume was greater in both S1 trials (*Z* = −2.701, *p* = 0.007) and S2 trials (*Z* = −2.803, *p* = 0.005) relative to water trials, again with no difference observed between S1 and S2 trials (*Z* = −1.478, *p* = 0.139). These findings suggest that, on average, a greater duration and volume of retronasal airflow occurred during the response interval in S1 and S2 trials relative to water trials but that fluid condition did not influence the peak rate of airflow.

Although greater retronasal airflow observed prior to response initiation in S1 and S2 trials relative to water trials is partially attributable to the longer response latencies observed in S1 trials and S2 trials, potentially in the absence of variation in patterns of retronasal airflow, an inspection of respiration patterns across individual trials demonstrates that retronasal airflow often tracked behavioral responses on a trial-to-trial basis, occurring for a longer duration in trials with longer response latencies and terminating prior to or shortly after response occurrence in many cases. Thus, as shown in Figure 2C, while Participant B1 exhibited shallow retronasal pulses during the interval between fluid contact and response elicitation in all trial types, the pattern of pulses occurring in S1 and S2 trials differs noticeably from that occurring in water trials. The quantity of retronasal pulses prior to response initiation is greater in S1 and S2 trials (left panels), and alignment of respiration traces with behavioral responses (right panels) shows that prominent pulses occurring in water trials typically occurred after responding, while those occurring in S1 and S2 trials typically occurred just prior to responding, often temporally aligned with the response and with little retronasal airflow observed during the interval between responding and resumption of respiratory inhalation. Response latencies in S2 trials were typically longer than those in S1 trials, and numerous shallow pulses are evident in the longer latency trials. Similarly, as shown in Figure 2D, Participant B6 exhibited more prominent retronasal airflow during the response interval in S1 and S2 trials than in water trials, with retronasal pulses occurring reliably prior to response elicitation in those trials, and airflow attenuated following the response. In this participant, substantial pre-deglutitive airflow was evident across fluid conditions. While patterns of respiration varied substantially across individuals, retronasal airflow was more prominent during the response interval in juice trials compared to water trials in all participants. While this was primarily due to longer response latencies in juice trials for two participants (Supplementary Figure S1A,B), in the absence of substantial differences in respiration patterns across fluid conditions, retronasal pulses were temporally related to response occurrence in a substantial proportion of S1 trials, S2 trials, or both for 8/10 participants (Figure 2C,D; Supplementary Figure S2C–H). These findings suggest that the occurrence of retronasal airflow was often associated with the time course of the perceptual decision in the discrimination task.

## 4. Discussion

### 4.1. System Evaluation

The present study demonstrates the utility of a relatively inexpensive, consumer-grade BCI platform for remotely monitoring human respiratory and EMG signals. The system included a lightweight biosensing board (Cyton), which we integrated with a miniature MEMS-based pressure transducer to monitor intranasal air pressure. The Cyton board supports eight high-quality physiological differential amplifiers (expandable to 16 channels; ADS1299 ADC; Texas Instruments, Inc., Dallas, TX, USA), three of which we used to monitor EMG activity. In addition, we made use of three available general-purpose analog inputs to monitor the voltage output of the respiration pressure transducer and the timing of session stimuli (tone onset) and responses (fluid contact).

The OpenBCI graphic user interface (GUI) is available as an open source and is fully modifiable. We ran the GUI within the Processing3 integrated development environment (The Processing Foundation, Brooklyn, NY, USA) to edit and launch the GUI. Despite a number of interesting visualizations (e.g., fast Fourier transform, spectrogram, EEG headplot, focus) of EMG and EEG signals in the GUI, we found the analysis tools rather limited for our purposes and exported data to Matlab for all analyses. One benefit of using an open-source platform to collect data is easy access to data, which is not possible without a subscription or software purchase for some other low-cost biosensing systems.

Several researchers have demonstrated the utility of consumer-grade BCI systems in EEG recording [22,27,28], and it may be possible to use the present system in future research examining the relationship between human respiration patterns and olfactory epithelial [29] or bulbar [30] activity. The Cyton’s fixed sampling rate of 250 Hz was sufficient for our purposes, and the pressure transducer we utilized was sufficiently sensitive to permit the assessment of fine patterns of nasal air pressure. However, as mentioned by Rashid et al. [22], this sampling rate could limit the examination of high frequency (e.g., gamma band) EEG activity. A significant benefit of the Cyton board was that it supported communication with the data acquisition processor (MacBook Pro; Apple, Inc., Cupertino, CA, USA) via Bluetooth, enabling participants to move freely, untethered by amplifier wires. In the present experiments, the board was attached to the participant’s arm, but it could be easily worn as a halter or belt attachment. Although the quality of the recording was satisfactory, dropped signals occurred sporadically when the distance between the data acquisition processor and the biosensing board was greater than approximately 2 m, separated by an interior wall. This was not a significant impediment to data collection in our setting, though, and investigation revealed that this distance could be increased to approximately 5 m when the processor and board were positioned in the same room.

### 4.2. Physiological and Behavioral Findings

We utilized the system to characterize patterns of retronasal airflow during the performance of an oral fluid discrimination task, and we showed that the chemical properties of an orally sampled stimulus may alter airflow dynamics in a manner that optimizes retronasal olfaction. We demonstrated an increased likelihood of retronasal airflow during the interval immediately following oral fluid intake regardless of fluid identity and during a later interval limited to trials involving discrimination of perceptually similar juice solutions. Examination of the magnitude of respiration signals during the response interval revealed that the volume and cumulative duration of retronasal airflow were greater in trials involving the juice solutions than in those involving water, but that peak airflow rate did not differ across fluid conditions. Further, while the magnitude and duration of individual post-deglutitive pulses (“swallow breaths”) did not differ between solutions, an inspection of respiration patterns across individual trials revealed that these pulses were more likely to occur prior to responding in juice trials and were often temporally related to behavioral responding. Importantly, pre-deglutitive airflow was present in most trials and was sufficient for accurate discrimination of similar solutions in approximately 20% of trials. Collectively, these findings suggest that sensorimotor processes regulate retronasal airflow to optimize the delivery of volatiles to the olfactory epithelium during oral fluid discrimination and that discrimination of perceptually similar oral fluids may be accomplished by prolonging the interval over which pulses with an optimal airflow rate occur rather than by varying the airflow rate per se.

The present findings suggest participants altered the duration and the volume of retronasal airflow but not the peak rate of airflow in discrimination trials, likely requiring retronasal olfaction for accurate performance. Indeed, most participants exhibited an overall low rate of outward airflow during fluid sampling, punctuated with one or more low-amplitude retronasal pulses, and in contrast to our predictions, with no apparent variation in amplitude (or vigor) across all three fluid conditions. Further, no differences were observed in the peak airflow rate on post-deglutitive pulses across fluid conditions. The observed pattern suggests that participants sampled retronasal airflow for a longer duration on the more perceptually challenging juice trials than in water trials but that retronasal pulses were not more vigorous in those trials. These findings resemble those reported by Sobel et al. [31], demonstrating that participants sniffed longer with the low flow rate nostril, but did not adjust sniff flow rate, in order to maintain orthonasal perceptual detection thresholds when the high flow rate nostril was occluded. This is not to say that airflow rate was unimportant in the present study since it was often noticeably lower during fluid sampling than during respiration. This finding is evident in several individual cases, including that illustrated in Figure 2C, where pre-response retronasal pulses occur more often and over a longer period in juice trials compared to water trials but were of a similar rate and lower in amplitude (lower airflow rate or vigor) than those occurring following the resumption of respiratory inhalation seconds later. The trend of altered retronasal airflow rates during fluid sampling compared to during respiration was evident in 6/10 cases (5 higher, 1 lower), suggesting that an optimal airflow rate may have contributed to accurate discrimination performance. Indeed, the velocity of airflow across the olfactory epithelium would be expected to influence the deposit of volatile molecules with different sorption rates, ultimately influencing the activation of central olfactory networks involved in odor perception. A body of research has long explored the impact of orthonasal airflow rate during the sniff cycle on sensory and perceptual features of olfactory processing [32,33,34], and one area for potential future investigation will be to examine the relationship between retronasal airflow rate and sensory and perceptual features of flavor quality and intensity.

The specific pattern of respiratory airflow has been shown to be modulated by a wide range of influences, such as sniffing, chewing and deglutition [11,23,35]. Subtle alterations in the pattern of retronasal airflow may contribute to the perceptual discrimination of orally presented stimuli that stimulate both gustatory and olfactory sensory pathways. Within the brainstem, the nucleus of the solitary tract (nucleus tractus solitarius; NTS) has been shown to play a role in the regulation of both respiration [36] and deglutition [37]. In addition, the dorsal nucleus of the NTS has long been known to be a primary target for gustatory sensory input, with ascending thalamocortical projections that are critical for taste perception (reviewed in [38]). However, Escanilla et al. [39] demonstrated that, in the rat, simultaneous presentation of olfactory and gustatory stimuli modulates the activity of individual cells in the NTS compared to gustatory stimuli alone, suggesting that these cells may serve as a site of convergence for gustatory and olfactory input. Thus, local connections within subdivisions of the NTS and between the NTS and other medullary nuclei may provide a means by which gustatory and olfactory inputs alter respiratory patterns to optimize retronasal airflow and thereby facilitate flavor discrimination, particularly between stimuli with similar chemical properties.

Although the methods utilized in the present study permit measurement of overall nasal airflow during fluid consumption, the position of the cannula tips within the nasal vestibule did not permit assessment of airflow within the dorsally-situated olfactory region. Computational fluid dynamics modeling of airflow within the canine nasal cavity suggests that sniffing may result in greater orthonasal airflow within the olfactory sensory region compared to during quiet breathing and that retronasal airflow between sniffs is minimal during exhalation [11]. As suggested by these authors, airflow over the olfactory region may be unidirectional during bouts of sniffing, which may enhance odorant uptake and cautions that airflow dynamics within the nasal cavity are not fully reflected in gross measures taken at the nares. Retronasal airflow dynamics within the nasal cavity during food or fluid consumption are not well understood, but evidence suggests that the anatomical structure of the oropharynx may result in the transportation of volatiles from the oral cavity to the nasal cavity during exhalation but not toward the lungs during inhalation [40]. The present study suggests that patterns of retronasal airflow during consumption are characterized by multiple bouts of pre-deglutitive and post-deglutitive airflow; however, additional work will be required to assess how such patterns may influence airflow and the deposition of volatiles within the olfactory sensory region.

The finding that deglutition apnea was shorter in duration during consumption of the sweet juice solutions than during consumption of water is in contrast to data reported by Leow et al. [41], who failed to show an impact of taste variation on apnea duration during consumption of flavored gelatin samples. One possible explanation for this discrepancy might relate to potential differences in the volume of consumed fluid across samples, which was neither manipulated nor measured in the present study. Hirst et al. [23] reported that deglutition apnea varied as a function of volume of a fluid bolus, with a significant increase in apnea duration with 20 mL water samples compared to 5 mL samples. Thus, it is plausible that differences in fluid volume consumed may have been a source of variability influencing apnea duration across fluid conditions.

The use of an automated fluid presentation system allowed participants to develop a repetitive behavioral pattern of active fluid intake. Although the topography and timing of respiration and fluid intake behavior varied across participants, each individual exhibited a generally consistent pattern across trials, which facilitated a comparison of retronasal airflow across stimulus conditions. The findings that behavioral response accuracy was poorer for S1 and S2 trials compared to water trials and that response latencies were longer in those trials suggest discrimination between S1 and S2 stimuli was more perceptually challenging than was the discrimination of either of these stimuli from water, which was likely detected rapidly based on taste profile alone. Thus, discrimination of the two juice solutions likely required additional processing consistent with their similar taste and aroma profiles. Although there is a possibility that the discrimination of the juice stimuli was influenced by properties detected within the oral cavity, such as sugar content or acidity, the finding that performance dropped to chance levels in participants wearing a nose plug suggests the involvement of retronasal olfactory processes in freely-breathing participants. It is notable that one participant wearing a nose plug performed at 76% accuracy in S1 trials (Supplementary Figure S1A), outperforming 1/13 freely-breathing participants. However, given that the mean response latency for this participant in S1 trials (5.40 s) was substantially greater than that of the poorest performing freely-breathing participant (1.51 s), and indeed greater than all other participants, it is unlikely that performance was influenced by oral cavity cues, which were available soon after fluid contact. Instead, it is likely that the elevated performance of this participant was due to chance variability or to incomplete restriction of nasal airflow. Thus, we interpret the behavioral findings to suggest that behavioral procedures encouraged the use of retronasal processes and were likely dependent on such processes for accurate performance. Future research may benefit from the use of “purely olfactory” stimuli (e.g., vanillin, phenylethyl alcohol, coumarin), which have been shown to be discriminable retronasally but not activate the oral cavity trigeminal system or to be discriminable based on oral cavity properties alone when presented to the mouth in the vapor phase [42,43].

## 5. Conclusions

In summary, the present study demonstrated the utility of a low-cost, open-source biosensing platform for monitoring low-frequency physiological signals associated with human olfactory behavior. The system enabled reliable characterization of fine patterns of retronasal airflow during the performance of an oral fluid discrimination task with perceptually similar juice solutions. Findings suggest that retronasal airflow is regulated by sensorimotor processes to optimize the delivery of volatiles to the olfactory epithelium during oral fluid sampling and that fine flavor discrimination may be accomplished by varying the duration of optimal retronasal airflow rate.

## Figures and Tables

**Table 1:** Comparison of deglutition measures and retronasal airflow measures across oral fluid conditions. Air pressure voltage traces were extracted for the first period of deglutition apnea and the first retronasal post-deglutitive air pulse in each correct trial, as well as for the response interval between fluid contact and the behavioral response. Peak rate was calculated as the maximum voltage value during each interval, and airflow volume was calculated as the area above the session-wide mode (no airflow). Voltage is represented in arbitrary units of magnitude. ^#^ Denotes significant main effect of fluid condition (Friedman test, *p* < 0.05). * Denotes significant difference from water stimulus condition (Wilcoxon signed-rank test, *p* < 0.0167). No post hoc comparisons conducted between S1 and S2 conditions were statistically significant.

		Deglutition Apnea		Post-Deglutitive Airflow			Response Interval Airflow		
Stimulus		Duration (s)	Latency (s)	Duration (s)	Peak Rate (Voltage)	Volume (Voltage ^2^)	Duration (s)	Peak Rate (Voltage)	Volume (Voltage ^2^)
Water	Median	0.67	2.15	0.99	165.79	3647.33	1.21	213.12	3317.86
	Mean	0.77	2.05	0.96	163.03	3921.63	1.24	215.48	3954.41
S1	Median	0.60 *	1.76	1.07	172.09	3644.65	1.60 *	213.88	5092.11 *
	Mean	0.63	1.77	1.04	175.33	4078.26	1.81	209.98	5839.57
S2	Median	0.62 *	1.70	0.88	177.44	2578.91	1.84 *	214.18	5976.99 *
	Mean	0.61	1.80	1.04	177.62	3857.45	1.98	210.53	6716.29
Friedman	*N*	10	10	10	10	10	10	10	10
	*χ2*	12.2	4.2	0.8	4.2	0.8	16.8	1.4	12.6
	*p*	0.002 ^#^	0.122	0.670	0.122	0.670	0.000 ^#^	0.497	0.002 ^#^

## Data Availability

Data supporting reported results can be found at https://github.com/grahamcousens/Characterization-of-retronasal-airflow-patterns (accessed on 25 August 2022).

## Supplementary Materials

The following supporting information can be downloaded at: https://www.mdpi.com/article/10.3390/s22186817/s1, Supplementary Findings: Behavioral Performance; Figure S1: Behavioral performance in the oral fluid discrimination task; Figure S2: Nasal airflow during the performance of the oral fluid discrimination task.
