# Supplementary material for: Characterization of Retronasal Airflow Patterns during Intraoral Fluid Discrimination Using a Low-Cost, Open-Source Biosensing Platform
Source: Sensors (Basel). 2022 Sep 9;22(18):6817. doi: 10.3390/s22186817 (PMC9505993; doi:10.3390/s22186817)
Supplement: Supplementary file 1 [file sensors-22-06817-s001.zip › sensors-1873729-SI.pdf]

sensors

MDPI

Supplementary Materials

Behavioral Performance

Thirteen of the sixteen participants completed the session, with a median accuracy of 94.23% correct trials and a median response latency of 2.76 s in the correct trials. As shown in Figure S1, performance varied across oral fluid conditions, with reduced accuracy and longer response latencies in the S1 and S2 trials compared to the water trials. One-sample Wilcoxon signed-rank tests demonstrated that the participants performed well above chance (50% correct) on S1 trials (median of 94.12% correct; $Z = -3.203$; $p = 0.001$) and S2 trials (median of 88.89% correct; $Z = -3.209$; $p = 0.001$). However, accuracy was reduced on the S1 and S2 trials in comparison to the water trials (Figure S1A), as suggested by a Friedman test showing a significant main effect of oral fluid conditions on the percentage of correct trials ($\chi 2 = 10.5$, $p = 0.005$). Post hoc Wilcoxon signed-rank tests revealed a significant decrease in accuracy in both the S1 trials ($Z = -2.481$; $p = 0.013$) and S2 trials ($Z = -3.123$; $p = 0.002$) relative to the water trials, but no significant difference between the S1 and S2 trials ($Z = -1.435$, $p = 0.151$). This decrease in accuracy in the S1 and S2 trials was accompanied by longer response latencies (Figure S1B), as suggested by a significant main effect of oral fluid conditions ($\chi 2 = 21.385$, $p < 0.001$). Post hoc tests revealed that the response latencies were significantly slower in both the S1 (median of 3.01 s; $Z = -3.181$; $p = 0.001$) and S2 trials (median of 3.25 s; $Z = -3.180$; $p = 0.001$) relative to the water trials (median of 1.84 s), but that the difference in the latencies between the S1 and S2 trials was marginally non-significant ($Z = -2.201$, $p = 0.028$). Thus, despite accurate discrimination performance overall, participants took longer to complete trials involving juice solutions and were less accurate in responding in those trials.

In order to confirm the fact that the discrimination between the S1 and S2 stimuli was dependent on retronasal olfaction rather than on oral cavity properties, such as sugar content or acidity, four additional participants performed the task while wearing a nose clip (Liquid Comfort, Speedo International Ltd., Nottingham, UK), substantially reducing the retronasal transport of volatiles to the olfactory epithelium. The median response latency (1.20 s) and response accuracy (100% correct) in the water trials during these sessions were similar to those observed in the absence of a nose plug. However, the median response latencies in the S1 (4.89 s) and S2 (4.59 s) trials were substantially longer for these participants compared to freely breathing participants, and the median accuracies in the S1 (52.94% correct) and S2 (58.33% correct) trials were similar to chance. These findings suggest that oral cavity cues are sufficient for rapidly discriminating between water and the two juice solutions used here, but that the retronasal transport of volatiles contributes to accurate discrimination between perceptually similar juice solutions.

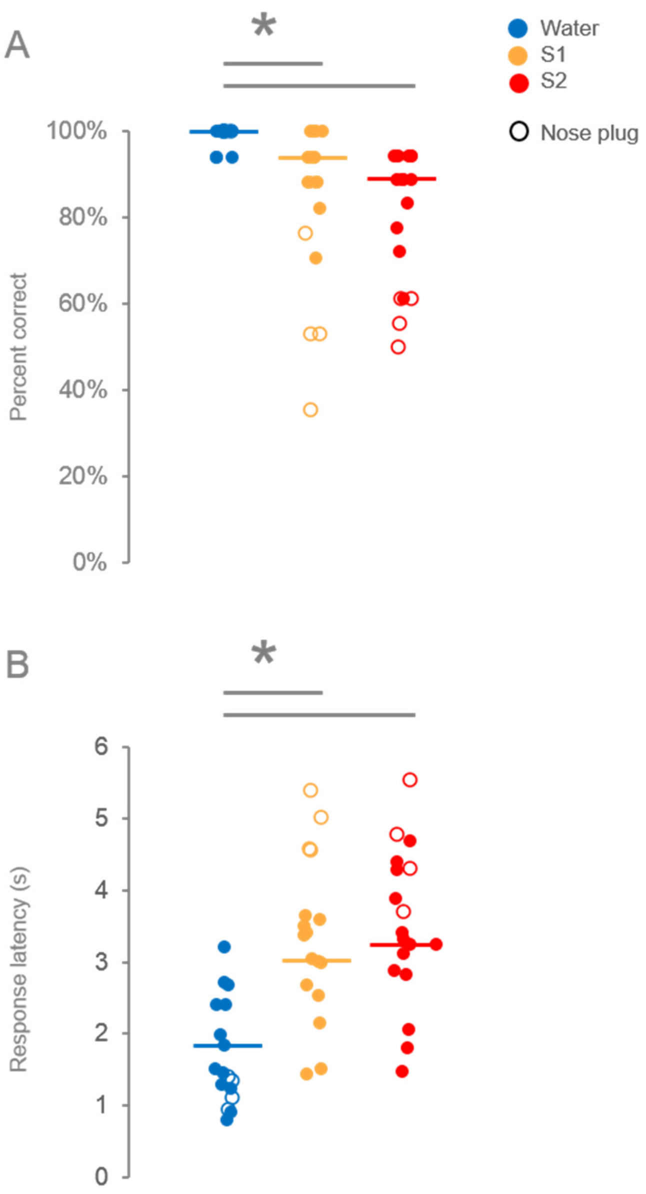

Figure S1. Behavioral performance in the oral fluid discrimination task. (**A**) Percentage of correct trials for each participant in the water, S1, and S2 trials. (**B**) Mean response latency for each participant in the water, S1, and S2 trials. Closed circles reflect the performance of freely breathing participants ($n = 13$), for whom respiration signals were recorded; open circles reflect the performance of participants ($n = 4$) wearing nose clips to restrict retronasal air flow. Horizontal bars indicate the sample medians of the freely breathing participants. * denotes a significant difference from the water stimulus condition (Wilcoxon signed-rank test, $p < 0.0167$). No post hoc comparisons conducted between the S1 and S2 conditions were statistically significant.

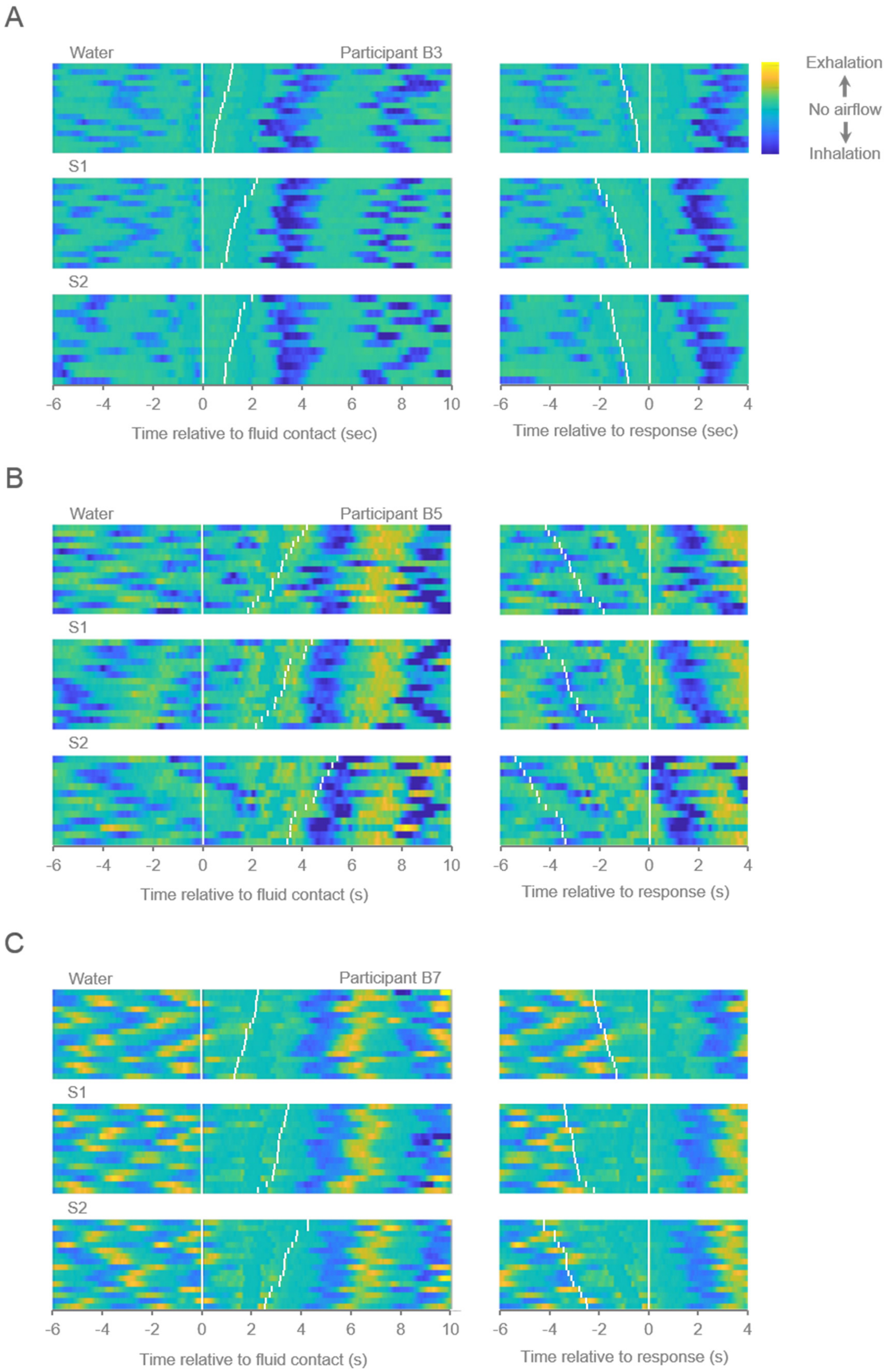
A
Water
Participant B3
S1
S2
-6 -4 -2 0 2 4 6 8 10
Time relative to fluid contact (sec)
-6 -4 -2 0 2 4
Time relative to response (sec)
Exhalation
No airflow
Inhalation
B
Water
Participant B5
S1
S2
-6 -4 -2 0 2 4 6 8 10
Time relative to fluid contact (s)
-6 -4 -2 0 2 4
Time relative to response (s)
C
Water
Participant B7
S1
S2
-6 -4 -2 0 2 4 6 8 10
Time relative to fluid contact (s)
-6 -4 -2 0 2 4
Time relative to response (s)

D

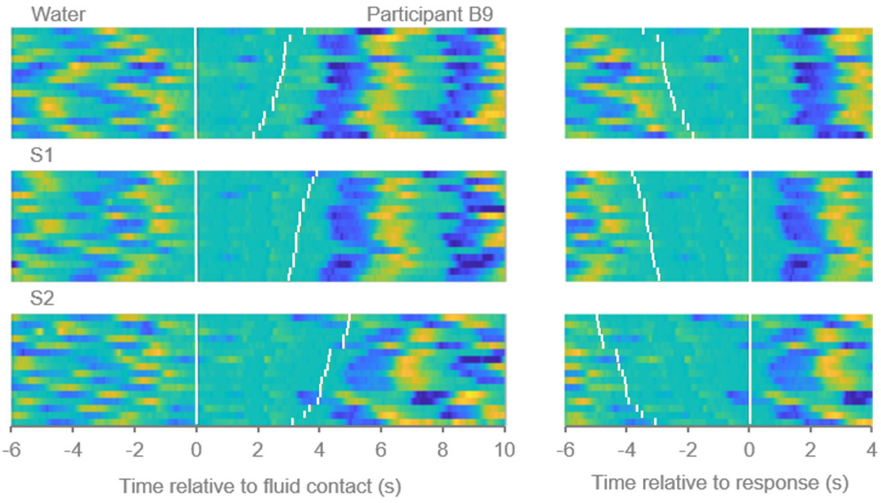
Water
Participant B9
S1
S2
-6 -4 -2 0 2 4 6 8 10
Time relative to fluid contact (s)
-6 -4 -2 0 2 4
Time relative to response (s)

E

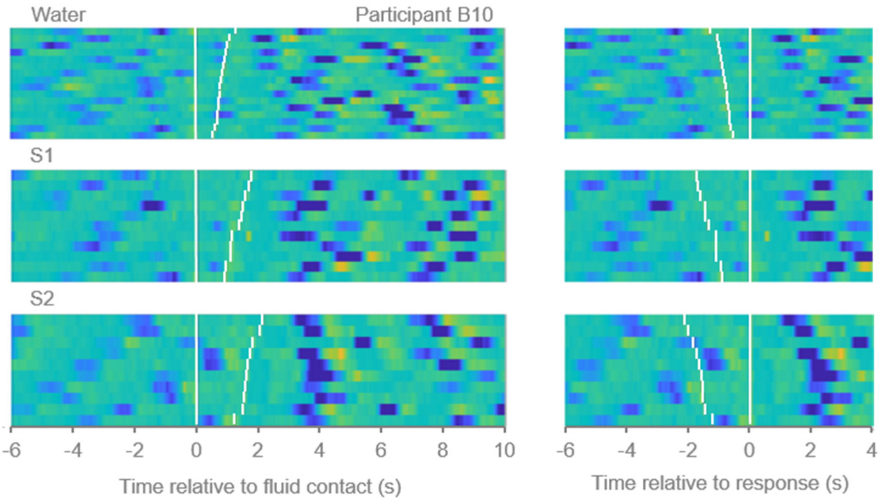
Water
Participant B10
S1
S2
-6 -4 -2 0 2 4 6 8 10
Time relative to fluid contact (s)
-6 -4 -2 0 2 4
Time relative to response (s)

F

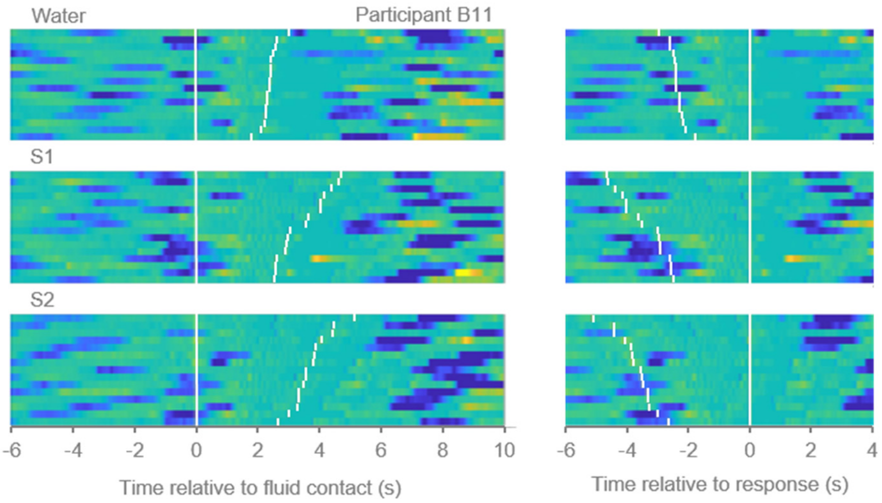
Water
Participant B11
S1
S2
-6 -4 -2 0 2 4 6 8 10
Time relative to fluid contact (s)
-6 -4 -2 0 2 4
Time relative to response (s)

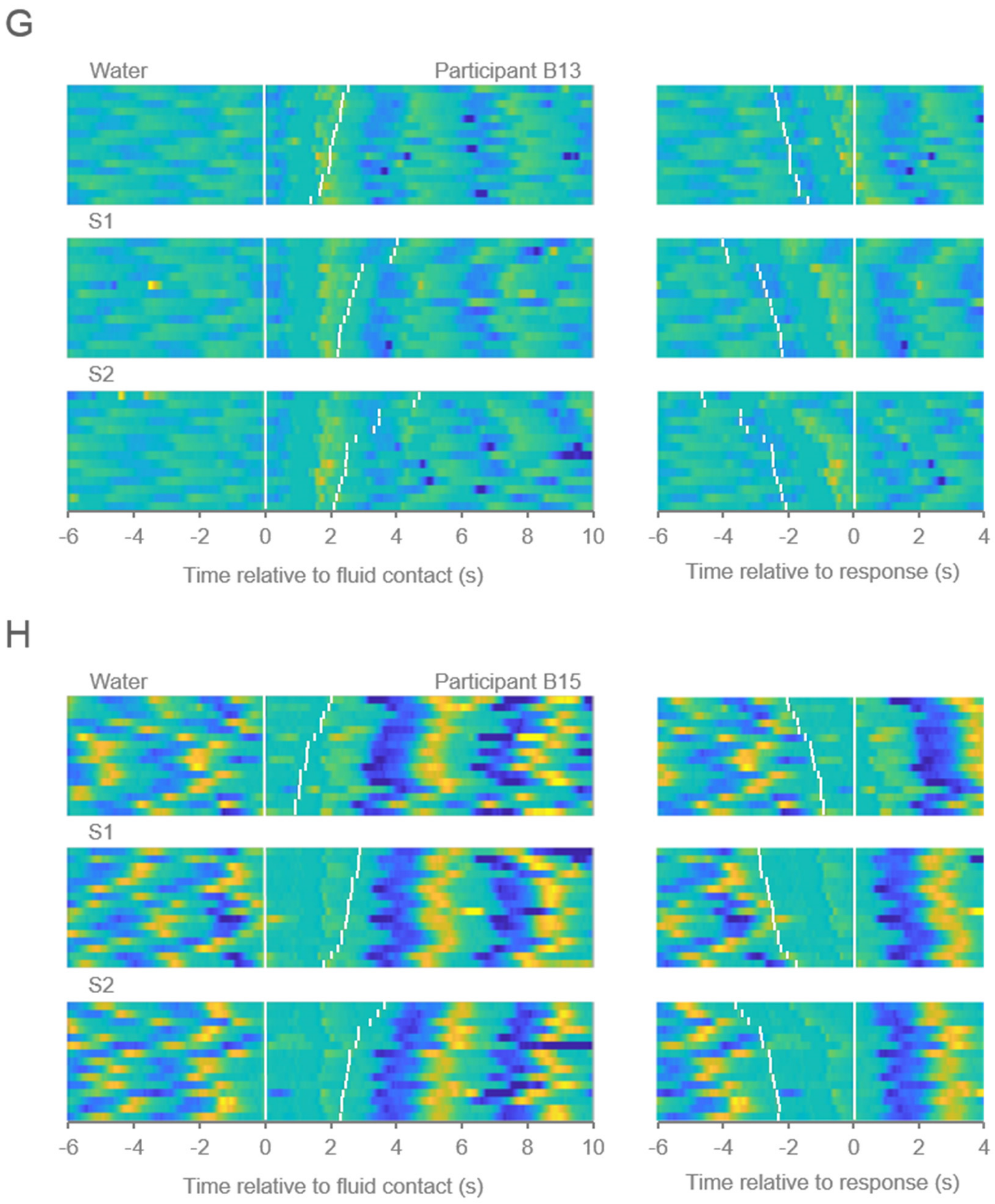

Figure S2. Nasal airflow during the performance of the oral fluid discrimination task (**A**–**H**). Contour plots of nasal air pressure for eight participants (B3, B5, B7, B9, B10, B11, B13, and B15) across all of the correct water, S1, and S2 trials. The vertical axis reflects the trials, which are sorted according to the response latency. The horizontal axis reflects the time (s) relative to the fluid contact (left panels) or response initiation (right panels) in each trial. White event markers reflect the time of occurrence of fluid contact (left marker) and the behavioral response (right marker) in each trial. The magnitude of retronasal (exhalation) or orthonasal (inhalation) airflow is reflected by the color intensity normalized for each participant, with the minimum voltage value pinned to the lowest color bar value (dark blue) and the session-wide mode voltage (no airflow) pinned to the middle color bar value (teal).
